# Modulation of the endocannabinoid system in viable and non-viable first trimester pregnancies by pregnancy-related hormones

**DOI:** 10.1186/1477-7827-9-152

**Published:** 2011-11-29

**Authors:** Anthony H Taylor, Mark Finney, Patricia MW Lam, Justin C Konje

**Affiliations:** 1 Endocannabinoid Research Group, Reproductive Sciences Section, Department of Cancer Studies and Molecular Medicine, University of Leicester, Leicester, UK

**Keywords:** Anandamide, cannabinoid receptor, endocannabinoid, miscarriage, PAPP-A, progesterone, β-hCG

## Abstract

**Background:**

In early pregnancy, increased plasma levels of the endocannabinoid anandamide (AEA) are associated with miscarriage through mechanisms that might affect the developing placenta or maternal decidua.

**Methods:**

In this study, we compare AEA levels in failed and viable pregnancies with the levels of the trophoblastic hormones (beta-human chorionic gonadotrophin (beta-hCG), progesterone (P4) and (pregnancy-associated placental protein-A (PAPP-A)) essential for early pregnancy success and relate that to the expression of the cannabinoid receptors and enzymes that modulate AEA levels.

**Results:**

The median plasma AEA level in non-viable pregnancies (1.48 nM; n = 20) was higher than in viable pregnancies (1.21 nM; n = 25; *P *= 0.013), as were progesterone and beta-hCG levels (41.0 vs 51.5 ng/mL; *P *= 0.052 for P4 and 28,650 vs 6,560 mIU/L; *P *= 0.144 for beta-hCG, respectively, but were not statistically significant). Serum PAPP-A levels in the viable group were approximately 6.8 times lower than those in the non-viable group (1.82 vs 12.25 mg/L; *P *= 0.071), but again these differences were statistically insignificant. In the spontaneous miscarriage group, significant correlations between P4 and beta-hCG, P4 and PAPP-A and AEA and PAPP-A levels were observed. Simultaneously, immunohistochemical distributions of the two main cannabinoid receptors and the AEA-modifying enzymes, fatty acid amide hydrolase (FAAH) and *N*-acylphosphatidylethanolamine-phospholipase D (NAPE-PLD), changed within both the decidua and trophoblast.

**Conclusions:**

The association of higher AEA levels with early pregnancy failure and with beta-hCG and PAPP-A, but not with progesterone concentrations suggest that plasma AEA levels and pregnancy failure are linked *via *a mechanism that may involve trophoblastic beta-hCG, and PAPP-A, but not, progesterone production. Although the trophoblast, decidua and embryo contain receptors for AEA, the main AEA target in early pregnancy failure remains unknown.

## Background

Successful implantation and maintenance of pregnancy occurs through a complex interaction between fetal and maternal tissues that involves several factors including the hormones estrogen and progesterone [1,2], human chorionic gonadotrophin [3-6], inhibin [5,7,8], pregnancy associated plasma protein (PAPP)-A [6,8,9], and a balance between type 1 T-helper (Th1) and type 2 T-helper (Th2) cytokines [10]. Although a unique immunological interplay is essential for the lack of rejection of the fetus by the mother, little is known about the precise factors responsible for the synchronous development of the embryo and the endometrium to ensure timely and successful implantation. Recent evidence from the murine model [11], and to a lesser extent, women undergoing IVF [12,13] has implicated the endocannabinoid system and in particular, the endogenous ligand, anandamide (AEA) in these processes. The endocannabinoid system consists of several natural ligands; i.e. AEA, 2-arachidonyl-glycerol (2-AG), *N*-oleoylethanolamine (OEA), *N*-palmitoylethanolamine (PEA) and virodhamine, their cognate and related receptors; i.e. cannabinoid receptors 1 (CB1) and 2 (CB2), the orphan receptor G-protein coupled receptor 55 (GPR55), peroxisome proliferator-activated receptor-α (PPAR-α) and the Ca^2+ ^channel vanilloid receptor 1 (TRPV1), and the enzymes that modulate the ligand concentrations; i.e. *N*-acyl transferase (NAT), *N*-acylphosphatidylethanolamine-phospholipase D (NAPE-PLD), fatty acid amide hydrolase (FAAH), diacylglycerol lipase (DAGL) and monoacylglycerol lipase (MAGL) [14,15].

Anandamide, originally isolated and characterised in 1992 [16], and four years after the cannabinoid receptors were identified [17], has been shown to be a key molecule that is important to the hormone-cytokine dialogue between the blastocyst and the endometrium in experimental animals, ensuring that the latter is 'tuned' to receive the former [11,18]. In such animals, alterations in the levels of AEA at the endometrium have resulted in failed implantation and/or failure to maintain pregnancy [19], suggesting that a decrease in the activation pathways for anandamide mainly *via *its G-protein-coupled receptor CB1 is essential for early pregnancy success. Indeed, experiments have demonstrated that female CB1 receptor knock-out mouse models not only exhibit impaired fertility but also suffer from impaired oviductal transport with resultant ectopic pregnancies [20], indicating that AEA signalling through the CB1 receptor is important in this aspect of mouse fertility.

In humans, such evidence at the level of the endometrium is lacking but the levels of the enzyme fatty acid amide hydrolase (FAAH), which is responsible for the catabolism of AEA into ethanolamine and arachidonic acid [21-26], measured at 6-8 weeks in asymptomatic pregnancies have been shown to be significantly lower in those that eventually spontaneously miscarry [13]. In addition, it has also been demonstrated that the plasma AEA levels at 6 weeks in women who had undergone IVF were significantly lower in those who achieved a viable pregnancy than in those who did not [12,13].

We have also shown that plasma levels of AEA in those presenting with a threatened miscarriage and an ultrasound confirmed viable pregnancy are significantly higher in those who subsequently spontaneously miscarried, compared to those who had live births [27]. All those with plasma AEA levels above 2 nM subsequently miscarried while 94% of those with plasma AEA levels <2 nM had live births. Whether these differences are similar in those who had already miscarried or had non-viable pregnancies is yet to be investigated.

Since progesterone, human chorionic gonadotrophin and pregnancy associated plasma protein (PAPP)-A, a trophoblast-derived protease that degrades the insulin-like growth factor binding proteins 4 and 5 [28] are involved in the regulatory processes responsible for the early maintenance of pregnancy, and progesterone has also been suggested to be involved in the regulation of AEA [29], we set out to examine whether there is (a) a difference in AEA levels in pregnancies which are confirmed to be either non-viable or viable and, (b) a relationship, between plasma AEA, serum progesterone (P4), human chorionic gonadotrophin (β-hCG) and PAPP-A in these two groups. Furthermore, we examined the expression and distribution of cannabinoid receptors 1 and 2 and the AEA metabolizing enzymes, FAAH and the key enzyme involved in AEA synthesis, NAPE-PLD, in the placenta and decidua of women having a spontaneous miscarriage and those undergoing induced abortions with an anti-progestin (mifepristone; RU486) and a prostaglandin (misoprostol) in comparison to the expression levels in normal pregnancy.

## Methods

### Patients and study design

This was a prospective cohort study of women attending the Early Pregnancy Assessment Unit (EPAU) of the University Hospitals of Leicester NHS Trust at the Leicester Royal Infirmary. All subjects gave signed, informed consent to take part in the study, which had approval of the Leicestershire and Rutland Research Ethics Committee. The research was sponsored by the local R&D office of the University Hospitals of Leicester NHS Trust.

A power analysis based on previous work of the Endocannabinoid Research Group and that of other published data of viable and non-viable pregnancies [13], with α = 0.05 and β = 0.8 showed that a minimum of 6 subjects was required in both the miscarriage and on-going pregnancy groups to allow a 40% change in plasma AEA levels to be observed with 80% power. Recruitment therefore continued until this minimum number was exceeded.

Asymptomatic women at 6-12 weeks amenorrhoea, presenting to the EPAU for a reassurance ultrasound because of a previous miscarriage, were recruited into the study prior to the ultrasound scan used to determine viability. For the viable pregnancies, only those with singleton pregnancies were included, because we were unsure of how multiple pregnancies would affect results. Blood samples were collected from the antecubital vein for the measurement of plasma AEA and various hormones. A total of 14 mL of blood was collected; 4 mL into an EDTA tube for AEA quantification and 10 mL into a plain tube for serum P4, β-hCG and PAPP-A measurement. The tubes containing the blood were placed on ice and transported to the research laboratory where plasma and sera were separated. Serum was stored at -80°C for later hormonal analysis while the plasma was processed for AEA quantification as described below.

For the immunohistochemical studies, products of conception were collected between 7 and 12 weeks gestation from women undergoing surgical termination (suction curettage; n = 60) and between 7 and 9 weeks for medical termination (600 mg mifepristone followed 36-48 hours later with 800-1200 mg misoprostol, n = 25) of pregnancy. All had an ultrasound scan examination to accurately date the pregnancies before termination as per the standard practice in the gynaecology unit. Samples were also collected from 16 spontaneous miscarriages, whose dates were determined by ultrasound scan. Collected tissues were transported on ice to the laboratory, where blood was washed away with sterile PBS and pieces of trophoblast and decidua dissected free with the aid of a dissecting microscope. Each piece was fixed in 10% neutral-buffered formalin for 4 days, dehydrated through 70% and 99% industrial methylated spirits (IMS), cleared with two changes of xylene and finally embedded in paraffin wax. This was performed in a LEICA ASP3000 automated vacuum tissue processor. Archival term placental tissues from women undergoing elective Caesarean section and full thickness uterine biopsies from the normal menstrual cycle acted as positive controls [30,31].

### Measurement of AEA

Plasma AEA was extracted using a modification of the method we previously described [32]. Briefly, the EDTA tube containing 4 mL of blood was immediately placed on ice and processed within 2 hours. After centrifugation at 1200 × *g *for 30 min at 22°C, 2 mL of plasma was transferred to a 7 mL glass Kimble scintillation vial (Fisher Scientific, Loughborough, UK) and 2.5 pmol of deuterium-labelled AEA (AEA-d_8_; Cayman Chemicals, Ann Arbor, MI, USA) was added. Plasma proteins were then precipitated by thoroughly mixing this mixture with an equal volume of ice-cold acetone followed by centrifugation at 1200 × *g *for 10 min at 22°C. The supernatant was transferred to a clean Kimble vial and subjected to a gentle stream of nitrogen gas for 40 min to evaporate the acetone. Lipid extraction was achieved by adding methanol:chloroform (1:2 vol; 2 mL) followed by centrifugation at 1200 × *g *for 10 min at 22°C. The lower chloroform layer was then recovered, dried under nitrogen gas and reconstituted in 80µl HPLC-grade acetonitrile. The reconstituted mixture was then injected into an Acquity UPLC in line with a Quattro Premier mass spectrometer (Waters Ltd., Elstree, UK) for ultra-high performance liquid chromatography-tandem mass spectrometry (UPLC-MS/MS) and measured as described [33].

All solvents and ammonium acetate used were of HPLC grade (Fisher Scientific, Loughborough, UK) and HPLC grade water was obtained using a water purification system (Maxima ELGA, ELGA, High Wycombe, UK).

### Hormone assays

Serum β-hCG and P4 were measured by the Biochemistry Department of the University Hospitals of Leicester NHS Trust using the ADVIA Centaur Immunoassay System (Bayer HealthCare LLC, Diagnostics Division; Tarrytown, NY, USA). Serum β-hCG was quantified using the ADVIA Centaur total β-hCG two-side sandwich immunoassay (Bayer HealthCare LLC, Diagnostics Division; Tarrytown, NY, USA) with a detection range of 2 mIU/mL to 1000 mIU/mL. If a level of >1000 mIU/mL was found, a dilution step was preformed and the assay repeated, allowing up to 100,000 mIU/mL to be detected. The intra- and the inter-assay coefficients of variation were 2.8%, and 2.9% respectively. For serum P4 measurement, the ADVIA Centaur progesterone competitive immunoassay using direct chemiluminescence (Bayer HealthCare LLC, Diagnostics Division; Tarrytown, NY, USA) was used with a detection range of 0.48 ng/mL to 190.8 ng/mL with intra- and inter-assay coefficient of variation of 5.3%, and 3.6% respectively.

Serum PAPP-A was measured using a commercially available ELISA kit (Gamma S.A., Liege, Belgium) according to the manufacturer's instructions with absorbance at 625 nm and 420 nm to determine the signal intensities of the product and background, respectively. The relative light intensities were measured on an Ascent Multiscan ELISA plate reader (Labsystems, Helsinki, Finland). The level of detection was 2-300 mg/L, and the inter- and intra-assay co-efficients of variation were 2.9% and 5.4%, respectively.

### Immunohistochemistry

Anti-CB1 and anti-CB2 rabbit polyclonal antibodies were obtained from Sigma-Aldrich Ltd. (Poole, Dorset, UK), whilst anti-FAAH rabbit polyclonal antibody was obtained from Alpha Diagnostics International, Inc., (San Antonio, TX) and anti-NAPE-PLD polyclonal antibodies were from Cayman Chemical Corp. (Ann Arbor, MI). Microwave antigen retrieval [34] was performed for the CB1, CB2 and NAPE-PLD studies as previously described [31,35,36], but was not required for the FAAH studies, because visual inspection of the staining intensities of representative samples with and without antigen retrieval were identical. The secondary goat anti-rabbit horseradish peroxidise conjugates were from Dako (Glostrup, Denmark), and the tyramide amplification and detection system was from PerkinElmer LAS (Beaconsfield, Buckinghamshire, UK). An ABC detection system (ABC Elite; Vector Laboratories, Peterborough, UK) was used in conjunction with 3,3'-diaminobenzidine (Vector Laboratories) to detect the presence of immunoreactive complexes for the anti-CB2, anti-FAAH and anti-NAPE-PLD antibodies, whereas the tyramide amplification system was substituted for ABC Elite for the detection of anti-CB1 complexes. The anti-FAAH antibodies were used at an optimal dilution of 1:2000 in PBS [137 mM NaCl, 9.2 mM Na_2_HPO_4_, and 1.46 mM KH_2_PO_4 _(pH 7.6)], whilst CB1 at 1:4000, CB2 at 1:500 and NAPE-PLD at 1:200 dilutions were created in TBS [500 mM Tris, 1.5 M NaCl (pH 7.6)]. For each antibody used, negative controls were performed using the same concentrations of rabbit IgG (for CB1, CB2 and NAPE-PLD; Vector Laboratories) and normal rabbit serum (for FAAH; Dako, Glostrup, Denmark). Sections were lightly counterstained with Mayer's Haematoxylin (Sigma-Aldrich Ltd., Poole, Dorset, UK), dehydrated through graded alcohols, cleared twice in xylene, and mounted in DPX mountant (BDH, Poole, Dorset, UK). To ensure consistency for histomorphometric analyses, all slides (60 surgical terminations, 25 medical terminations and 16 spontaneous miscarriage samples) were processed simultaneously in a single run for each antibody and both positive and negative control tissues compared with previous data [30,31].

### Histomorphometric analyses

The pattern of distribution of the positively stained cells was recorded as being absent (-), present (+), absent in some samples but present in others (-/+) or more intense (++) in the various cellular compartments of the first trimester placenta (cytotrophoblast, syncytiotrophoblast and mesenchymal core) and in the decidua for the entire dataset. The number of positively stained cells per field was assessed by the capture of 10 randomly selected fields per slide at 400× magnification [37] for 10 samples of surgical terminations, 10 medical abortions and 10 spontaneous miscarriages. The samples were matched for gestational age and were all from weeks 7 and 8 of gestation. Images were captured using an Axioplan microscope (Carl Zeiss, Herts. UK) and a DXC-151P colour video camera (Sony CCD/RGB) at the same setting with the light levels set at 6400K in the presence of daylight and neutral density filters. Immunostaining levels in the trophoblast layer (cytotrophoblast and syncytiotrophoblast) and in the mesenchymal core of the placenta and the entire decidua were obtained separately using an unbiased histoscore method inside the pixel count algorithm (version 9) contained within the Imagescope^® ^software package (Aperio Technologies Inc., Vista, CA, USA). Weakly stained areas were weighted 100, stained areas 200 and strong staining weighted 300; unstained areas and non-stained areas were weighted 0. Unstained sections were used as negative controls and sections treated with IgG/serum instead of immunised antibody/serum used for the baseline (non-stained) staining levels.

### Statistical analysis

Statistical analysis of the data was performed using GraphPad Prism version 5.00 for Windows (GraphPad Software, San Diego California USA, [38]). Data that did not follow a Gaussian distribution are expressed as medians and inter-quartile ranges (IQR) where appropriate, and comparisons between groups performed using Mann-Whitney U-test. Correlations between hormone measurements were made using Spearman's correlation. The histomorphometric data were normally distributed and so comparisons were made using one-way ANOVA with Bonferroni's *ad hoc *post-test. The level of significance was set at *P*<0.05.

## Results

### Hormone and anandamide measurements

A total of 45 women were recruited; 25 with viable pregnancies (viable group) and 20 with non-viable pregnancies (non-viable group). There were no differences in the age or BMI between the two groups, as shown in Table 1. The median plasma AEA levels in the non-viable group (1.48 nM) were significantly higher than those in the viable group (1.21 nM) at the time of ultrasound scan (*P *= 0.013).

**Table 1:** Patient Demographics and Hormonal Measurements

Parameter	Viable Pregnancy (N = 25)	Non-Viable Pregnancy (N = 20)	Significance
**Age**	29 (23-34)	27 (23-29.5)	*P *= 0.34
**BMI (kg/m^2^)**	23 (22-26)	24.5 (21.3-26)	*P *= 0.87
**AEA (nM)**	1.20 (0.92-1.39)	1.48 (1.01-2.34)	***P *= 0.013**
**P4 (ng/mL)**	51.5 (42-63.5)	41.0 (9.5-65.0)	*P *= 0.052
**β-hCG (mIU/mL)**	28650 (14172-38707)	6560 (386-49254)	*P *= 0.144
**PAPP-A (mg/L)**	1.82 (1.56-5.73)	12.25 (1.99-19.63)	*P *= 0.071

Age, body mass index (BMI), plasma AEA levels, serum progesterone (P4), β-hCG and PAPP-A levels are all show as median and (IQR). N = the number of patients in each group. Significance was calculated using Mann-Whitney U-test. Significant differences between viable and non-viable pregnancies are shown with a **bold ***P*-value.

Although serum P4 levels were lower in the non-viable (41 nmol/L) compared to the viable (51.5 ng/mL) group, this difference was not statistically different (*P *= 0.052). Similarly, although the levels of β-hCG were lower in the non-viable group (6560 mU/mL) compared to the viable group (28650 mU/mL), this also did not reach statistical significance (*P *= 0.144). The median serum PAPP-A levels in the non-viable group (12.25 mg/L) were approximately seven times those in the viable group (1.82 mg/L), but due to the large range of PAPP-A values obtained, this also was not statistically significant (*P *= 0.071).

The inter-relationship between plasma AEA levels and the levels of the hormones measured are shown in Table 2. There were no statistically significant correlations between plasma AEA levels and serum progesterone (r = 0.017; *P *= 0.926), AEA and β-hCG (r=-0.258, *P *= 0.162) and between AEA and PAPP-A (r = 0.35, *P *= 0.086) in the 45 patients. There was, however, a statistically significant negative correlation between plasma AEA levels and the length of gestation (r=-0.327, *P *= 0.028). There were statistically significant correlations between β-hCG and progesterone (r = 0.576; *P *= 0.001), between PAPP-A and P4 levels (r = 0.506, *P *= 0.027) and between PAPP-A and β-hCG (r = 0.512, *P *= 0.025).

**Table 2:** Spearman rank correlations between serum hormone and plasma AEA levels in women within the 1^st ^trimester of pregnancy.

All samples	P4			β-hCG			PAPP-A			AEA		
	r-values	*P*-values	n	r-values	*P*-values	n	r-values	*P*-values	n	r-values	*P*-values	n
**β-hCG**	0.576	***0.001***	**31**									
**PAPP-A**	0.506	***0.027***	**19**	0.512	***0.025***	**19**						
**AEA**	0.017	0.926	**31**	-0.258	0.162	**31**	0.351	0.086	**25**			
**Gestation**	0.045	0.809	**31**	0.338	0.063	**31**	-0.192	0.337	**27**	-0.327	***0.028***	**45**

Progesterone (P4) was measured in ng/mL, β-hCG was measured in mIU/mL, PAPP-A was measured in mg/L, AEA was measured in nM and gestation was measured in weeks. The Spearman co-efficients (r) were calculated using GraphPad Prism software and significant correlations are in ***bold***. The number of samples (n) is given for each correlation.

A further and separate analysis of the relationships between plasma AEA levels and PAPP-A, β-hCG and P4 in the viable and non-viable groups was undertaken (Table 3). In the viable group, there was a negative correlation between β-hCG and plasma AEA levels (r = -0.466, *P *= 0.051), although this did not achieve statistical significance it was close to our cut-off of *P*<0.05. There were also negative but statistically insignificant relationships between P4 and AEA and between PAPP-A and AEA (*P*>0.05). In the non-viable group, there was a statistically significant positive correlation between P4 and β-hCG (r = 0.739, *P *= 0.004), P4 and PAPP-A (r = 1.00, *P*<0.0001) and between AEA and PAPP-A (r = 0.697, *P *= 0.025). As expected β-hCG had a statistically significant positive correlation with gestation (r = 0.750, *P *= 0.003). Plasma AEA levels also had a negative correlation with gestational age (r=-0.475; *P *= 0.035). All of the other relationships were not statistically significant.

**Table 3:** Spearman rank correlations between serum hormone and plasma AEA levels in women within the 1^st ^trimester of pregnancy with viable and non-viable pregnancies.

	P4			β-hCG			PAPP-A			AEA		
	r-values	*P*-values	n	r-values	*P*-values	n	r-values	*P*-values	n	r-values	*P*-values	n
**Viable Pregnancies**												
**β-hCG**	0.391	0.109	**18**									
**PAPP-A**	0.266	0.404	**12**	0.427	0.167	**12**						
**AEA**	-0.247	0.323	**18**	-0.466	***0.051***	**18**	-0.004	0.990	**15**			
**Gestation**	-0.086	0.735	**18**	0.386	0.114	**18**	0.156	0.579	**15**	-0.246	0.236	**25**
**Non-viable Pregnancies**												
**β-hCG**	0.739	***0.004***	**13**									
**PAPP-A**	1.000	***<0.0001***	**4**	0.300	0.624	**5**						
**AEA**	0.401	0.175	**13**	0.052	0.865	**13**	0.697	***0.025***	**10**			
**Gestation**	0.490	0.089	**13**	0.750	***0.003***	**13**	-0.532	0.114	**10**	-0.475	***0.035***	**20**

Progesterone (P4) was measured in ng/mL, β-hCG was measured in mIU/mL, PAPP-A was measured in mg/L, AEA was measured in nM and gestation was measured in weeks. The Spearman co-efficients (r) were calculated using GraphPad Prism software and significant correlations are in ***bold***. The number of samples (n) is given for each correlation.

### Cannabinoid receptor and AEA modifying enzyme expression in the trophoblast

**Figure 1:**
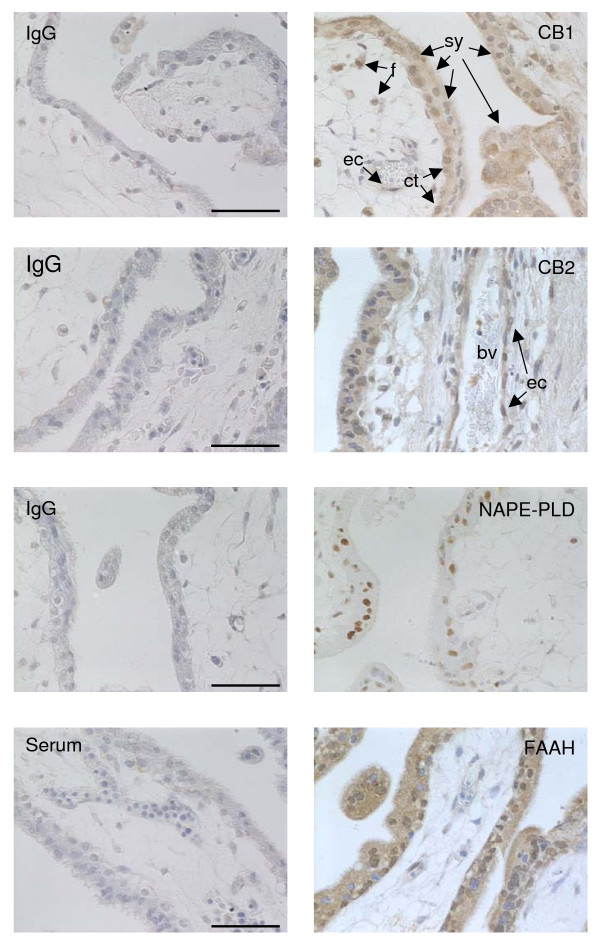
**Antibody specificity**. The CB1, CB2, NAPE-PLD and FAAH antibodies demonstrated specific staining to first trimester trophoblast. CB1 specific staining was visible in the syncytiotrophoblast (sy), cytotrophoblast (ct) and fibroblast (f) cell cytoplasm and to a lower intensity in the cytoplasm of the fetal endothelial cell (ec). Perinuclear and nuclear CB1 immunoreactivity was also observed in the cytotrophoblast layer, but only in the occasional syncytiotrophoblast (sy). CB2 immunoreactivity was also observed to be cytoplasmic, with immunopositive cells observed within the fetal blood vessels (bv), whereas NAPE-PLD immunoreactivity appeared almost exclusively in the nuclei of immunoreactive cells. FAAH immunoreactivity was both nuclear and cytoplasmic with differential expression of nuclear staining within the cytotrophoblastic layer. Photomicrographs are representative images taken from surgical terminations at weeks 7 and 8 of gestation. All images were captured at 400× magnification; bar = 50 μm. Respective rabbit IgG isotype or rabbit serum controls at equivalent concentrations are shown in the left panels.

**Figure 2:**
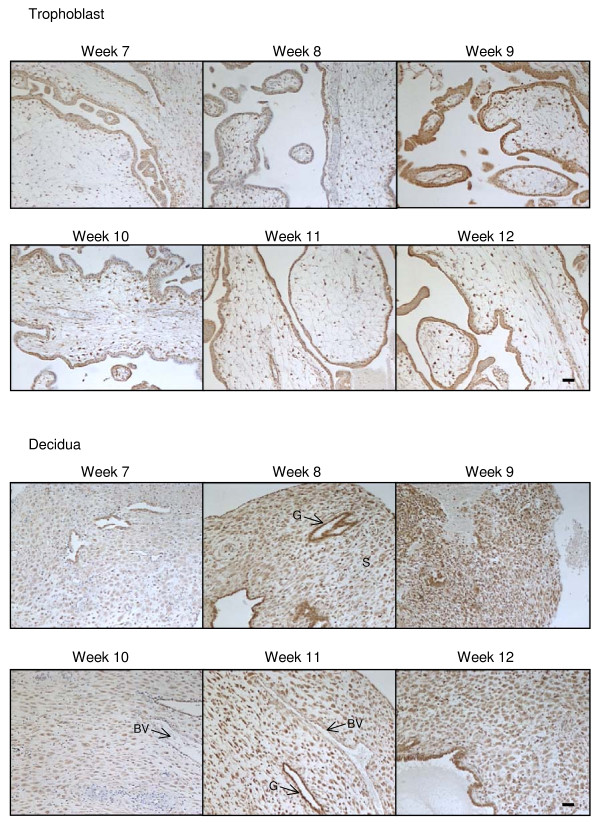
**The effect of gestation on CB1 immunoreactivity**. CB1 immunostaining is present throughout the first trimester in both the trophoblast (upper panels) and decidua (lower panels). In the trophoblast, the majority of the staining was confined to the cytotrophoblast and syncytiotrophoblast layers, whereas intense immunoreactivity was observed in the decaying endometrial glands (G) and decidualised stromal fibroblasts (S) of the early decidua. By contrast, blood vessels (BV) had much lower staining intensities. Images were taken at 100× magnification and are representative of samples taken for each of the gestational dates indicated. Bar = 50 μm.

The expression patterns for CB1, CB2, NAPE-PLD and FAAH (Table 4) in the trophoblast of surgical terminations were similar to that reported [39-41] with immunoreactive CB1 visible in the syncytiotrophoblast and cytotrophoblast layers, in the mesenchymal core and the endothelial cells of the blood vessels but not in fetal blood cells or infiltrating maternal plasma cells nor in the vascular smooth muscle cells (Figure 1). In tissues from the surgical terminations, CB1 immunoreactivity in the syncytiotrophoblast layer diminished in intensity with advancing gestation, but did not disappear at gestation weeks 10 to 12, as previously reported (41; Figure 2).

**Table 4:** Immunohistochemical localisation and staining intensities of the various components of the endocannabinoid system in the first trimester trophoblast and decidua.

Antigen	Cell type	Surgical termination	Medical termination	Spontaneous miscarriage
**CB1**	Syncytiotrophoblast Cytotrophoblast (Histoscore) Mesenchymal core (Histoscore) Decidua (Histoscore)	+/++ ++ (161.0 ± 4.77) - (3.3 ± 0.40) ++ (66.6 ± 3.69)	+ + **(113.9 ± 7.48)** + **(15.5 ± 2.25)** ++ (**36.4 ± 5.57**)	+ + (**79.8 ± 7.91**)* - (**8.8 ± 0.96**)* + (**15.0 ± 2.04**)*
**CB2**	Syncytiotrophoblast Cytotrophoblast (Histoscore) Mesenchymal core (Histoscore) Decidua (Histoscore)	+ + (96.5 ± 11.01) -/+ (9.0 ± 1.96) + (14.8 ±1.59)	+/++ + (114.3 ± 16.40) -/+ (14.3 ± 2.4) +/++ (**68.8 ± 8.04**)	++ ++ (**233.5 ± 5.27**)* ++ (**223.5 ± 5.27**)** +/++ (**78.7 ± 6.06**)^ns^
**NAPE-PLD**	Syncytiotrophoblast Cytotrophoblast (Histoscore) Mesenchymal core (Histoscore) Decidua (Histoscore)	-/+ + (9.4 ± 2.77) - (1.7 ± 0.56) -/+ (4.9 ± 0.51)	+/++ + (**32.0 ± 2.45**) - (2.0 ± 0.23) + (**11.2 ± 1.62**)	-/+ +/++ (**14.2 ± 2.68**)^ns^ - (1.3 ± 0.38)^ns^ -/+ (4.6 ± 0.69)*
**FAAH**	Syncytiotrophoblast Cytotrophoblast (Histoscore) Mesenchymal core (Histoscore) Decidua (Histoscore)	+ -/+ (45.5 ± 7.08) ++ (42.0 ± 6.02) + (13.9 ± 2.34)	+/++ ++ (**127.8 ± 6.26**) + (22.7 ± 5.03) -/+ (23.2 ± 6.38)	+ + (**105.2 ± 7.74**)^ns^ - (**2.6 ± 0.59**)* ++ (**55.3 ± 4.00**)**

Scoring system: (-) = absent; (+) = staining visible; (-/+) = staining occasionally absent;

(++) = stronger staining. The histoscore value for each antigen is shown as (mean ± sem) under each tissue that it represents. Each histoscore consists of 10 random fields/slide, for 10 matched samples each of surgical termination, medical abortion and spontaneous miscarriages taken from weeks 7 and 8 of gestation, as described in the Materials & Methods section. Data in bold are significantly different to respective surgical termination tissue and *P<0.01;**P<0.001; One-way ANOVA with Bonferroni's *ad hoc *post-test when compared to the medical abortion group.

**Figure 3:**
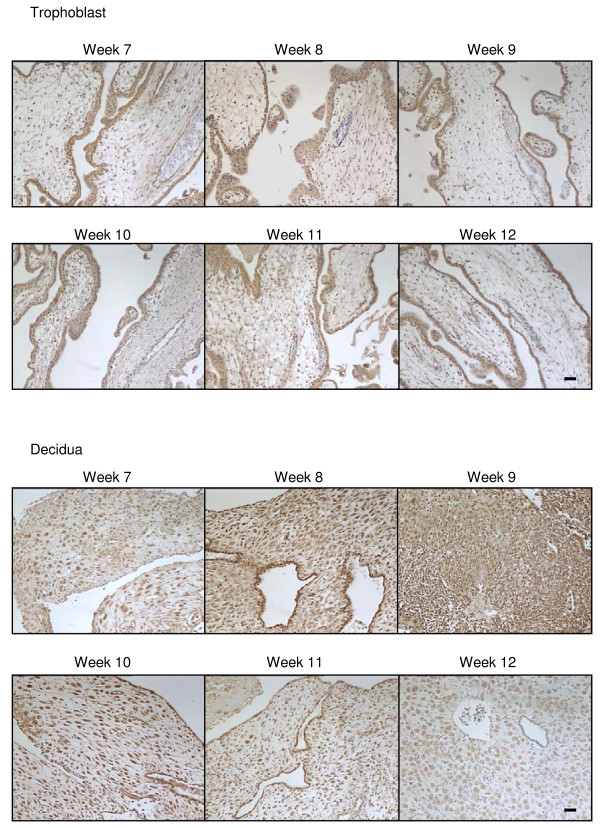
**The effect of gestation on CB2 immunoreactivity**. CB2 immunostaining is present throughout the first trimester in both the trophoblast (upper panels) and decidua (lower panels). In the trophoblast, the majority of the staining was confined to the cytotrophoblast and syncytiotrophoblast layers and infiltrating maternal plasma cells. Similar to the CB1 immunostaining, intense CB2 immunoreactivity was observed in the decaying endometrial glands and decidualised stromal fibroblasts. The intensity of decidual cell staining increased and reach a peak around week 10. By contrast blood vessels had much lower staining intensities. Images were taken at 100× magnification and are representative of samples taken for each of the gestational dates indicated. Bar = 50 μm.

Like CB1 expression, CB2 immunoreactivity of surgical terminations was detected in both the syncytiotrophoblast and cytotrophoblast layers, in the mesenchymal core and the endothelial cells of the blood vessels but not in fetal blood cells or infiltrating maternal plasma cells nor in the vascular smooth muscle cells (Figure 1) and the intensity of CB2 immunoreactivity in the syncytiotrophoblast remained constant throughout this gestational period and through various stages of the first trimester (Figure 3), in contrast to the immunoreactive staining patterns for CB1.

**Figure 4:**
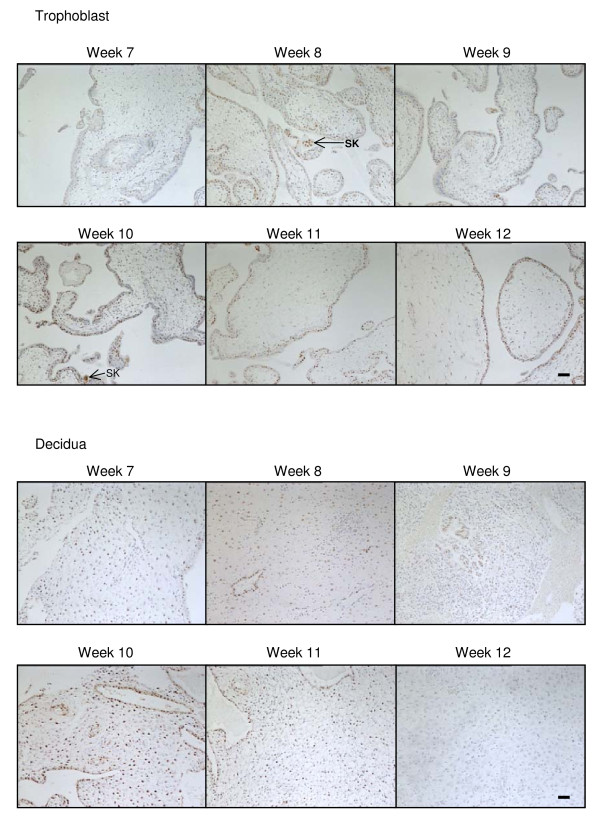
**The effect of gestation NAPE-PLD immunoreactivity**. NAPE-PLD immunostaining is present throughout the first trimester in both the trophoblast (upper panels) and decidua (lower panels). In the trophoblast, the majority of the staining was confined to the cytotrophoblast and syncytiotrophoblast layers, with the staining being preferentially observed in the cytotrophoblast layer when compared to the syncytiotrophoblast layer. Intense nuclear staining was occasionally observed in syncytial knots (SK) whilst only the occasional mesenchymal fibroblast showed NAPE-PLD immunoreactivity. Intense immunoreactivity was observed in some of the decidualised stromal fibroblasts. The intensity of decidual cell staining was variable and reached a maximum at week 10 but was essentially absent in the stromal fibroblast by week 12. Images were taken at 100× magnification. Bar = 50 μm.

Immunoreactive NAPE-PLD was detected in cells that formed the cytotrophoblast layer and the occasional syncytiotrophoblast cell and was primarily identified to be present in the nuclei of immunopositive cells (Figure 1). NAPE-PLD immunoreactivity was occasionally observed in the nuclei of the mesenchymal core of the developing villi, but essentially this tissue was negative for NAPE-PLD immunoreactivity (Figure 1). NAPE-PLD immunoreactivity in the cytotrophoblast layer of surgical terminations remained constant throughout the first trimester (Figure 4).

**Figure 5:**
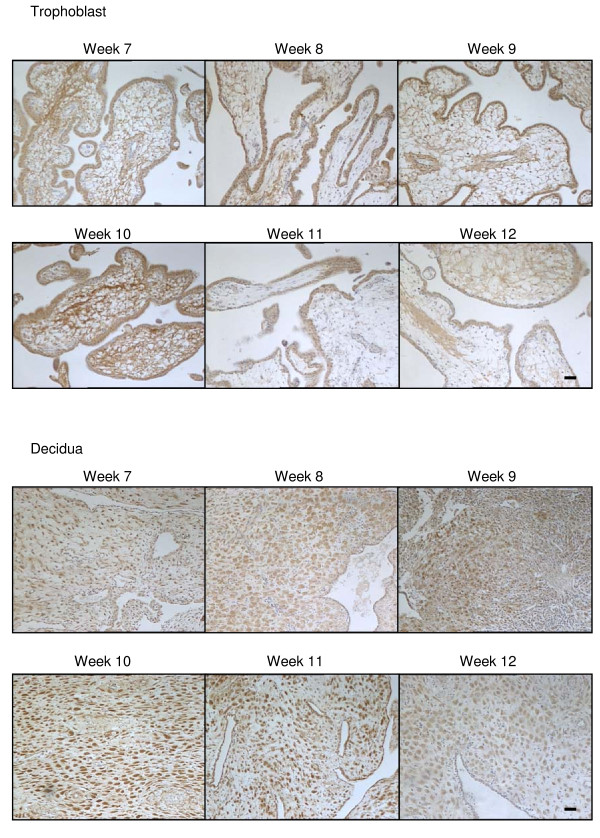
**The effect of gestation on FAAH immunoreactivity**. FAAH immunostaining is present throughout the first trimester in both the trophoblast (upper panels) and decidua (lower panels). In the trophoblast, the majority of the staining was confined to the cytotrophoblast and syncytiotrophoblast layers, although significant immunoreactivity was observed in the mesenchymal core. The intensity of FAAH immunoreactivity throughout the trophoblast diminished in weeks 11 and 12 of gestation. Intense immunoreactivity was observed in the decidualised stromal fibroblasts and decaying endometrial glands. The intensity of decidual cell staining increased and reached a peak around week 10 and was diminished by 12. Images were taken at 100× magnification. Bar = 50 μm.

Immunoreactive FAAH in surgical terminations was noted predominantly throughout the cytoplasm of cells with some nuclear staining (Figure 1), in keeping with the membranous and intracytoplasmic vesicular localization of FAAH, except in extravillous trophoblasts where intense nuclear staining was often observed. This was similar to that observed in the invasive trophoblasts of recurrent miscarriages [39]. Cytoplasmic/membranous FAAH protein was observed in both the cytotrophoblast and syncytiotrophoblast layers and the mesenchymal core of the developing villi and in infiltrating maternal plasma cells (Figure 5), but not in the fetal cells (Figure 1). Immunoreactive FAAH was detected in all 1st trimester syncytiotrophoblast and cytotrophoblast layers between weeks 7 and 12 (Figure 5) and the intensity of staining increased gradually in the syncytiotrophoblast layer of surgical terminations between the 7th and the 10th week of gestation (Figure 5). By the 11th week, FAAH immunoreactivity at the syncytiotrophoblast layer had diminished to the point where it was barely detectable within large parts of the trophoblast, an effect that persisted into the 12th week (Figure 5).

**Figure 6:**
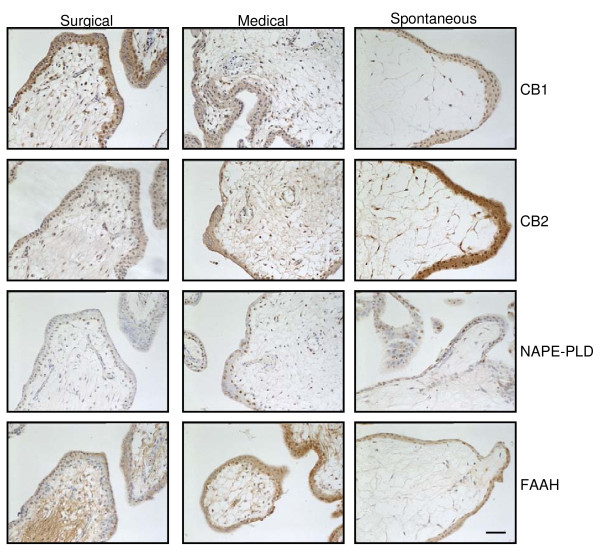
**The effect of medical abortion and spontaneous miscarriage on the localisation of endocannabinoid system in first trimester trophoblast**. Images are representative of 60 surgical and 25 medical terminations, and 16 spontaneous miscarriages. Spontaneous abortion is characterised by a marked decrease in the expression of CB1, increased CB2 and FAAH expression, whilst NAPE-PLD expression is unaffected. By contrast, medical termination of pregnancy is characterised by a small decrease in CB1 expression, a small increase in NAPE-PLD expression and a marked increase in cytotrophoblastic FAAH expression that does not occur in spontaneous miscarriage, which is characterised by a marked decrease in mesenchymal FAAH expression. Images were taken at 200× magnification. Bar = 50 μm. Surgical termination of pregnancy was used as the control condition.

Comparison of the CB1, CB2, FAAH and NAPE-PLD staining patterns in tissues from medical terminations and spontaneous miscarriages showed that CB1 expression in the trophoblast layer decreased in both types of tissue when compared to that from surgical terminations (Figure 6; Table 4). By contrast, CB2 immunoreactivity increased in tissues from medical terminations and significantly more so in the tissues from the spontaneous miscarriage group (Figure 6; Table 4). NAPE-PLD immunoreactivity was primarily observed in the nucleus of the cytotrophoblast of the surgical termination group. Within the medical termination group, NAPE-PLD immunoreactivity was not only observed in the nucleus of the cytotrophoblast, but also in the syncytiotrophoblast and in the stromal fibroblasts of the villi (Figure 6; Table 4). NAPE-PLD staining was similarly present in the cytotrophoblast and syncytiotrophoblast layers in the spontaneous miscarriage group, but not in the stromal fibroblasts. The levels of immunoreactive FAAH in the cytotrophoblast layers were significantly increased in both the medical termination and spontaneous miscarriage groups, with a more diffuse cytoplasmic staining pattern observed in the syncytiotrophoblast layer of the miscarriage samples when compared to the surgical termination samples (Figure 6; Table 4).

### Cannabinoid receptor and AEA modifying enzyme expression in the decidua

CB1 immunoreactivity was observed in the decidua throughout the first trimester surgical termination samples, with little or no modulation from weeks 7 to 12 (Figure 2). Within the decidual cell, CB1 immunoreactivity was observed primarily within the cytoplasm and plasma membrane and was absent from the nucleus of most cells (Figure 2). The staining was variable and 'patchy' with clusters of decidual cells demonstrating similar staining intensities that differed from adjacent areas of decidua (Figure 2). In the endothelial cells of larger capillaries, CB1 immunoreactivity was also observed in the nucleus.

CB2 immunoreactive protein was also observed in the surgical termination samples, but at a much lower level when compared to CB1 immunoreactivity (Figure 3). As was observed for CB1 immunoreactivity, CB2 immunoreactivity was also observed primarily within the cytoplasm and plasma membrane and was absent from the nucleus of most cells. It was also absent from endothelial cells of the larger capillaries (Figure 3). CB2 immunoreactivity gradually increased in the decidual cell during the first trimester (Figure 3) with the greatest immunoreactivity observed in weeks 9/10 of gestation.

NAPE-PLD immunoreactivity was observed in the decidua throughout the first trimester, although the staining was not uniformly distributed (Figure 4). In line with the CB1 staining pattern in the decidual cells, NAPE-PLD immunoreactivity was variable and 'patchy' with clusters of decidual cells demonstrating similar staining intensities that differed from adjacent areas of decidua (Figure 4). Where the decidual cell was immunopositive, intense nuclear staining was observed. The endothelial cells were also immunopositive demonstrating nuclear staining (Figure 4).

FAAH immunoreactivity was observed in the decidua at levels equivalent to that observed for CB2 immunoreactivity (Figure 5). Staining in the decidual cell was located in the cytoplasm and occasionally observed in the nucleus. The cells surrounding the larger capillaries showed a similar level of FAAH immunoreactivity in the cytoplasm, whilst endothelial cells appeared to be immunonegative (Figure 5). The expression of FAAH in the first trimester decidua gradually increased reaching a peak at weeks 9/10 (Figure 5).

**Figure 7:**
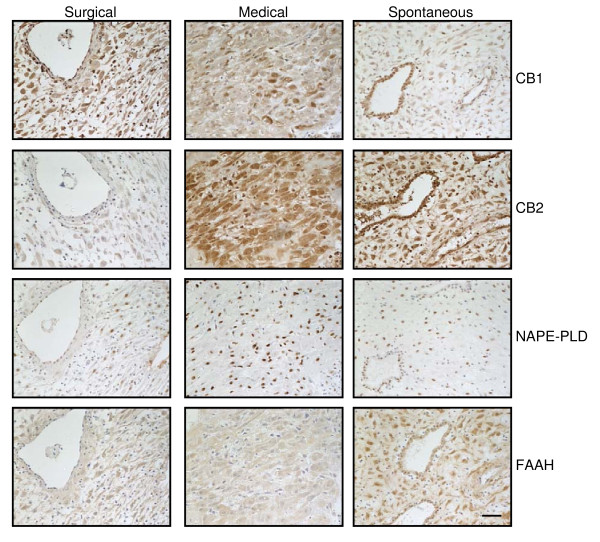
**The effect of medical abortion and spontaneous miscarriage on the localisation of the endocannabinoid system in first trimester decidua**. Images are representative of 60 surgical and 25 medical terminations, and 16 spontaneous miscarriages. Medical termination of pregnancy is characterised by an increase in the expression of CB2 and NAPE-PLD protein whilst overall CB1 expression is decreased and FAAH expression is unaffected. By contrast, spontaneous miscarriage is also characterised by a marked increase in CB2 and FAAH expression and a decrease in CB1 expression that exceeds that of medical termination samples. Images were taken at 200× magnification. Bar = 50 μm. Surgical termination of pregnancy was used as the control condition.

Comparison of the CB1, CB2, FAAH and NAPE-PLD staining patterns of medical terminations and spontaneous miscarriages indicated that CB1 expression in the decidua increased in individual cells of the medical terminations, but decreased in the spontaneous miscarriage group (Figure 7; Table 4). Histoscore analyses indicated that CB1 immunoreactivity decreased in both the medical termination and spontaneous miscarriage groups (Table 4). By contrast, CB2 immunoreactivity increased in the deciduas of both the medical termination and in the spontaneous miscarriage samples (Figure 7; Table 4) similar to that observed in the equivalent trophoblast (Figure 6; Table 4). The levels of immunoreactive FAAH were significantly increased in the spontaneous miscarriage group but not in the medical termination group, with a more diffuse cytoplasmic staining pattern observed in the medical termination deciduas when compared to the surgical terminations, whilst there was clear nuclear staining in the spontaneous miscarriage group (Figure 7; Table 4). NAPE-PLD immunoreactivity was increased in both the medical termination and in the spontaneous miscarriage groups, with more intense nuclear NAPE-PLD observed in the medical termination when compared to either the surgical termination or the spontaneous miscarriage samples (Figure 7; Table 4). There was no significant change in NAPE-PLD staining levels in the spontaneous miscarriage tissue when compared to the surgical termination group, except that staining was exclusively nuclear in the spontaneous miscarriage group, whereas some weak cytoplasmic immunoreactivity was observed in the surgical termination group (Figure 7; Table 4).

## Discussion

This study demonstrates that plasma AEA levels are elevated in women with non-viable first trimester pregnancies when compared to the levels in confirmed viable pregnancies. The levels in viable pregnancies were similar to those previously reported by Maccarrone *et al*. [13] and our group [27,32] and also similar to those reported in the luteal phase of the menstrual cycle [32,33,42], affirming the suggestion that for successful implantation, plasma AEA levels need to be maintained at a low level during both the implantation window [42] and early pregnancy development [12,32].

In the mouse, a significant fall in AEA and a rise in FAAH are essential at the implantation site compared to the inter-implantation site for successful implantation and early pregnancy maintenance [18,20]. The observations of Maccarrone *et al*. [43] and Habayeb *et al*. [27] suggest that in humans, peripheral mononuclear cell FAAH levels and the levels of AEA behave in a similar way to the local uterine endocannabinoid changes reported in the mouse uterus [44]. The findings of raised plasma AEA concentrations in non-viable pregnancies at the time of presentation suggests that an aberration in the endocannabinoid system might have occurred resulting in a disruption of the normal implantation and developmental process of the fetal-maternal interface, thus causing miscarriage. Precisely when this might have occurred is uncertain but since the levels of AEA in non-viable pregnancies are much lower than those we reported in women presenting with a threatened miscarriage and a viable pregnancy who subsequently miscarried [27], we believe that this may represent the AEA level returning to a 'baseline' following a peak at the time of fetal demise. The AEA levels found in these women were close to those seen during the follicular phase of the menstrual cycle [42,45] suggesting a preparation for the recommencement of the menstrual cycle.

Serum progesterone and β-hCG levels were similar to those reported in the literature [1,3,5,9,46-48]. As expected, serum progesterone levels were significantly lower in the non-viable group. These levels have been used to help in the diagnosis and management of failed pregnancies and more recently, pregnancies of unknown location [1,2,47-50]. Although median β-hCG levels were 4.4 times higher in the viable group, this difference was not statistically significant. Various studies have reported similar observations [3,5,6,8] and it is thought that the absence of significant differences is due largely to variations in the timing of the sample collection. For example, it is likely that some of our samples would have been taken immediately after the pregnancy changed from a viable to non-viable state and in others they would have been taken days later, when β-hCG levels would have already fallen significantly. Furthermore, it is known that as a predictor of pregnancy failure β-hCG is poor, with a sensitivity and specificity of only around 80%, and wide variations have been seen between studies when defining a threshold level for viability at a specific gestation [3].

The lack of a correlation between AEA and either serum β-hCG or progesterone in the cohort as a whole could easily be interpreted to mean that plasma AEA levels are linked to early pregnancy failure through a mechanism independent of these factors. The lack of a correlation between AEA and progesterone, however, is at odds with the suggestion of Maccarrone *et al*., that low P4 levels were associated with high plasma AEA levels, through a mechanism that involves the direct stimulation of FAAH (the main enzyme that degrades AEA) in peripheral mononuclear cells [13,29] and so we were therefore surprised by the lack of correlation between P4 and AEA levels in the present study, although a larger sample could possibly demonstrate the significant correlation we anticipated. At present, it is not clear how rapidly the levels of these biomarkers return to normal after miscarriage and if, as may be the case, one biomarker is cleared faster than the other, a previously existing correlation may thus be absent. Therefore, by combining the viable and non-viable groups to perform the correlations, we might have inadvertently hidden any correlations in the viable data and *vice versa*. Re-examining our data under the discrete groups of viable and non-viable pregnancies (Table 3) then revealed an almost significant negative correlation between AEA and β-hCG in the viable group and a significant correlation between β-hCG and P4 only in the non-viable group as would be expected based on published literature [9,51,52]. The serum levels of progesterone and PAPP-A were perfectly correlated, although the number of samples assayed was relatively small. There was also a correlation between plasma AEA levels and PAPP-A levels, but only in the non-viable pregnancies and a negative correlation between length of gestation and plasma AEA levels in the non-viable group, but not in the viable group.

A third possibility to explain the apparent discrepancies in the correlation analyses is the possibility that genetic abnormalities in the fetus may alter the expression of the enzymes controlling the levels of these biomarkers. Nevertheless, these data suggest that a complex interplay is involved between these factors and early pregnancy success.

Although PAPP-A levels were increased in the non-viable group these values did not reach statistical significance. A decrease in PAPP-A levels is now considered to be a marker of problems in pregnancy [6,8], and this discrepancy may be due to small numbers involved (because some measurements were below the limit of detection and so did not contribute to the analysis), or due to the fact that the pregnancies had already failed [53-55]. There was, however, a strong correlation between P4, β-hCG and PAPP-A levels in both viable and non-viable pregnancies, suggesting a causal link in the production of each of these factors. At present, the factors involved in the production of PAPP-A are unclear and although a number of factors have been implicated in its generation, P4 is not one of them [6,8,56]. The positive correlation between AEA and PAPP-A might also indicate a causal link between these two factors. There are two main possibilities here. Firstly, there is a pregnancy 'master regulator' that controls the expression of both AEA and PAPP-A, or secondly, that these two factors regulate each other. Currently, there is insufficient data to support either of these possibilities.

The finding of an association between raised plasma AEA and non-viable pregnancies could mean either that either a disruption in the endocannabinoid system led to a disruption of the normal implantation and developmental process, causing miscarriage or that a failed pregnancy, for whatever reason, is associated with a disruption in the endocannabinoid system in favour of a higher AEA level. Which of these possibilities is the more likely is uncertain. By investigating the expression patterns of the endocannabinoid system in the trophoblast and decidua, we hoped to further elaborate on some of these possibilities.

Although the trophoblast, the endometrium, and the embryo all contain CB1 and CB2 receptors, NAPE-PLD and FAAH, we are inclined to conclude that the trophoblast appears not to be the main target for the action of AEA in early pregnancy failure due to the lack of correlation between the levels of AEA and either β-hCG, P4 and PAPP-A. During early pregnancy, the placenta participates in the production of these three hormones and if AEA was having a detrimental effect on the developing trophoblast, then the levels of these hormones would be expected to be inversely modulated in relation to AEA levels. Furthermore, it would be anticipated that the plasma AEA levels would also be reflected at the local level by alterations in the expression of the AEA modulating enzymes NAPE-PLD and FAAH, such that either NAPE-PLD levels increased, whilst FAAH levels decreased or remained constant [57], or NAPE-PLD levels remain constant along with decreased FAAH levels. Such changes would thus cause the local AEA concentrations to increase. The staining patterns for these two enzymes actually showed that NAPE-PLD remained constant, whilst FAAH levels increased. These changes would result in decreased local AEA levels in the placenta should the enzymes be fully activated. The nuclear localisation of NAPE-PLD in trophoblast and in the decidua was surprise as AEA is variously reported as being synthesised 'on-demand' from membrane precursors and as such is considered to be an integral component of the plasma membrane [14,15]. The possibilities here are that (a) the NAPE-PLD protein identified by this antibody is inactive because it is localised in the nucleus, (b) that synthesised AEA is not made 'on-demand', but is made and sequestered within the cell, or (c) the NAPE-PLD antibody used in this study is not specific for NAPE-PLD and detects an alternative protein. The first possibility was not tested in this study and could still be correct, the second possibility is supported by recent evidence that AEA (being highly lipophilic) is transported around the cell on fatty acid binding proteins [58,59] and so AEA could be synthesised in sites other than the plasma membrane and store in adiposomes and the third possibility could also be true in that the antibody used does not detect the NAPE-PLD protein but some other irrelevant protein, but is unlikely, since we have recently demonstrated nuclear staining of NAPE-PLD in the rat placenta and decidua using a different source of NAPE-PLD antibody [60,61], suggesting that, in the pregnant uterus at least, NAPE-PLD protein is located in the nucleus. Similar nuclear NAPE-PLD staining was observed in the normal menstrual cycle and in the normal human ovary suggesting that NAPE-PLD is indeed expressed in the nucleus [31,36].

In contrast to the static expression of NAPE-PLD and increased expression of FAAH, there were reciprocal expressions of CB1 and CB2 in the trophoblast during the transition from the viable to non-viable state [62], with CB2 expression increasing in the trophoblast. These data suggest that AEA signalling through trophoblastic CB2 could mediate trophoblast demise similar to the effect that Δ^9^-tetrahydrocannabinol has on human trophoblast cell lines, again through the CB2 receptor [63]. Recent immunohistological staining patterns for CB1, CB2, FAAH, NAPE-PLD and the 2-AG modulating enzymes, MAGL and DAGL show similar patterns of expression in the mature rat placenta, with decreased CB1 expression at the end of gestation (unpublished observations). These data, with those of Acone *et al*., [57] are supportive of reduced CB1 signalling and increased FAAH expression in the placenta as being important not only in spontaneous and recurrent miscarriage [39], but also in the process of labour, at the end of pregnancy [57].

Furthermore, the increased expression of CB1 in the decidua of medical terminations and spontaneous miscarriages is totally in keeping with the recent evidence that modulation of CB1 receptor expression in the rat decidua occurs naturally and AEA induces apoptosis in the decidua through this receptor isoform [60,64]. Additionally, the demonstration that AEA modifying enzyme distribution in the decidua changes to support local accumulation of anandamide, i.e. NAPE-PLD expression similar to that found in the rat [61] suggest that AEA acts at the level of the local endometrial-embryo interface in the human too, and therefore primarily on the decidua. These data suggest that the decidua is the main target for AEA action during early pregnancy, although the trophoblast may also be a target.

## Conclusions

Our data confirm that there is an interaction between the components of the endocannabinoid system and the fetal-maternal interface and we believe that further studies of this interaction may eventually lead to the identification of potential areas for intervention to reduce the incidence of spontaneous miscarriages or new therapeutics for pregnancy termination.

## Competing interests

Professor Konje has a patent for the use of AEA in the detection of preterm labour. All other authors have no potential conflicts of interest.

## Authors' contributions

AHT and JCK conceived and designed the study. The recruitment and data collection were undertaken by AHT and MF. The samples were processed by AHT, MF and PMWL and the data analysed by AHT and MF. AHT, MF and JCK prepared the draft manuscript and the study was supervised by AHT and JCK. JCK is the guarantor of the paper and confirms that he has seen and has access to all the data. He takes full responsibility for the conduct of the study and controlled the decision to publish. All authors read and approved the final manuscript.
